# Institutional Notes and News

**Published:** 1911-06-17

**Authors:** 


### Institutional Notes and News.

**Admiral Togo at the Dreadnought.**

Admiral Togo, accompanied by Sir Frederick Fisher and Admiral Dundas, visited the Dreadnought Hospital at Greenwich on Tuesday afternoon. He was conducted through the building by Mr. P. J. Michelli, C.M.G., the secretary, and examined the arrangements made for the accommodation of Japanese sailors. It will be remembered that the hospital contains a bed endowed by the Emperor of Japan.

**The Royal Eye Hospital Dinner.**

Lord Decies presided this year at the dinner which was held last week in aid of the Royal Eye Hospital, Southwark. In the course of his speech, at its close, he said that a great deal of work remained to be done in preventing blindness among children. During the last two years, owing to the increase in the number of patients and a decline in subscriptions, the hospital had accumulated a debt of between £1,500 and £2,000. The administration was carried out on the most economical lines. Sir W. Collins said that the King's Hospital Fund had recognised the great work they were doing by making them a grant. There were those who thought that the National Insurance Bill, which every one approved and no one completely understood, might result in reducing the amount of public support given to voluntary hospitals, while increasing the calls upon the useful work which they accomplished. Among those present were Catherine Lady Decies, Mr. G. Brookbank James, Mr. Matthew Clark, Sir W. J. Collins, senior honorary surgeon, and Mr. E. Easton, secretary.

**Norwich Eye Infirmary and the Charity Commissioners.**

The proposed new order by the Charity Commissioners for the regulation of the Norfolk and Norwich Eye Infirmary and the Norfolk and Norwich Hospital, to which objections must be sent in on or before June 24, may be summarised as follows: The scheme shall come into operation on the date of the issue of a certificate by the Charity Commission that a building suitable for use as a new infirmary for diseases of the eye has been built. The future building with the site and appurtenances shall be an endowment of the Norfolk and Norwich Eye Infirmary. Until an adequate out-patients' department to be used in connection with the infirmary has been provided at the hospital the old infirmary buildings may be retained on the customary lines as an out-patients' department. Subject to these provisions, the trustees of the old infirmary shall sell the building, site, and appurtenances, with the approval of the Commissioners, and invest the proceeds in trust for the charity. The committee, to be appointed annually, shall consist of nine persons, of whom five were subscribers to the funds of the Eye Infirmary and four governors of the Norfolk and Norwich Hospital. The secretary and matron of the Norfolk and Norwich Hospital shall be ex officio secretary and matron of the Eye Infirmary. The committee out of the income of the Eye Infirmary shall pay to Mr. S. N. Berry, the present secretary, a pension of £15, and shall continue the present pension of £26 a year to Mrs. Rix.

